# Characterization of transgenic mouse lines for labeling type I and type II afferent neurons in the cochlea

**DOI:** 10.1038/s41598-019-41770-5

**Published:** 2019-04-03

**Authors:** Pankhuri Vyas, Jingjing Sherry Wu, Adrian Jimenez, Elisabeth Glowatzki, Paul Albert Fuchs

**Affiliations:** 1 The Center for Hearing and Balance, Otolaryngology-Head and Neck Surgery, Johns Hopkins University School of Medicine, Baltimore, MD 21205 USA; 2 Department of Neuroscience, Johns Hopkins University School of Medicine, Baltimore, MD 21205 USA; 3 Present Address: Department of Neurobiology, Harvard Medical School, Boston, MA 02115 USA

## Abstract

The cochlea is innervated by type I and type II afferent neurons. Type I afferents are myelinated, larger diameter neurons that send a single dendrite to contact a single inner hair cell, whereas unmyelinated type II afferents are fewer in number and receive input from many outer hair cells. This strikingly differentiated innervation pattern strongly suggests specialized functions. Those functions could be investigated with specific genetic markers that enable labeling and manipulating each afferent class without significantly affecting the other. Here three mouse models were characterized and tested for specific labeling of either type I or type II cochlear afferents. *Nos1*^*CreER*^ mice showed selective labeling of type I afferent fibers, *Slc6a4-GFP* mice labeled type II fibers with a slight preference for the apical cochlea, and *Drd2-Cre* mice selectively labeled type II afferent neurons nearer the cochlear base. In conjunction with the *Th*^*2A-CreER*^ and *CGRPα-EGFP* lines described previously for labeling type II fibers, the mouse lines reported here comprise a promising toolkit for genetic manipulations of type I and type II cochlear afferent fibers.

## Introduction

Spiral ganglion neurons (SGNs) receive inputs from hair cells, mechanoreceptors of the cochlea, to encode acoustic information into action potentials that travel into the central nervous system (CNS). SGNs are divided into two major groups based on their morphology and cochlear innervation pattern. Type I SGNs are larger diameter, myelinated neurons that constitute ~95% of the total auditory nerve fibers. They send a single dendrite to contact one inner hair cell (IHC). The remaining 5% are smaller diameter, unmyelinated type II afferent fibers that contact numerous outer hair cells (OHCs) as they spiral hundreds of microns towards the cochlear base^1,2^. Type I SGNs are responsible for encoding the salient parameters of sound^3^. Type II SGN function remains an area of active inquiry, with recent studies supporting a role in signaling tissue damage^4,5^.

Genetically engineered mouse lines that allow selective targeting and manipulation of specific neuronal groups are valuable tools for *in vivo* functional studies, fate-mapping during development, regeneration experiments and more. Since type II afferent fibers are few in number, small in caliber and unmyelinated, mouse genetic tools will be especially useful for defining their function *in vivo*. A variety of mouse lines have been described that label SGNs, for example: *Shh (Sonic hedgehog*)*-Cre*^6,7^, *Neurog1* (*Neurogenin1)-Cre*^8^, *Neurog1-CreER*^*T2*^ ^9^, *Bhlhb5-Cre*^8^, *PV (Parvalbumin)-Cre*^10^. However, Cre drivers such as these don’t distinguish type I from type II SGNs. The present work shows that all type I, but not type II SGNs express the enzyme neuronal nitric oxide synthase, making this a specific marker for future studies of type I SGNs.

Previous work has shown that tyrosine hydroxylase (TH) is preferentially expressed by apically-located type II afferents, while calcitonin gene related peptide alpha (CGRPα) is preferentially expressed by type II afferents in the cochlear base^11,12^. In the present work, two additional mouse lines are shown to specifically label type II SGNs, the serotonin transporter (SERT/Slc6a4) and a subunit of the dopamine receptor, *Drd2*. Furthermore, these expression patterns also reveal ‘tonotopic’ heterogeneity within the type II population. This strengthens the speculation that apical and basal type II afferents may serve distinct functions.

## Results

### Neuronal Nitric Oxide Synthase (*Nos1*^*CreER*^) specifically labels type I but not type II afferent neurons in the cochlea

**Figure 1:**
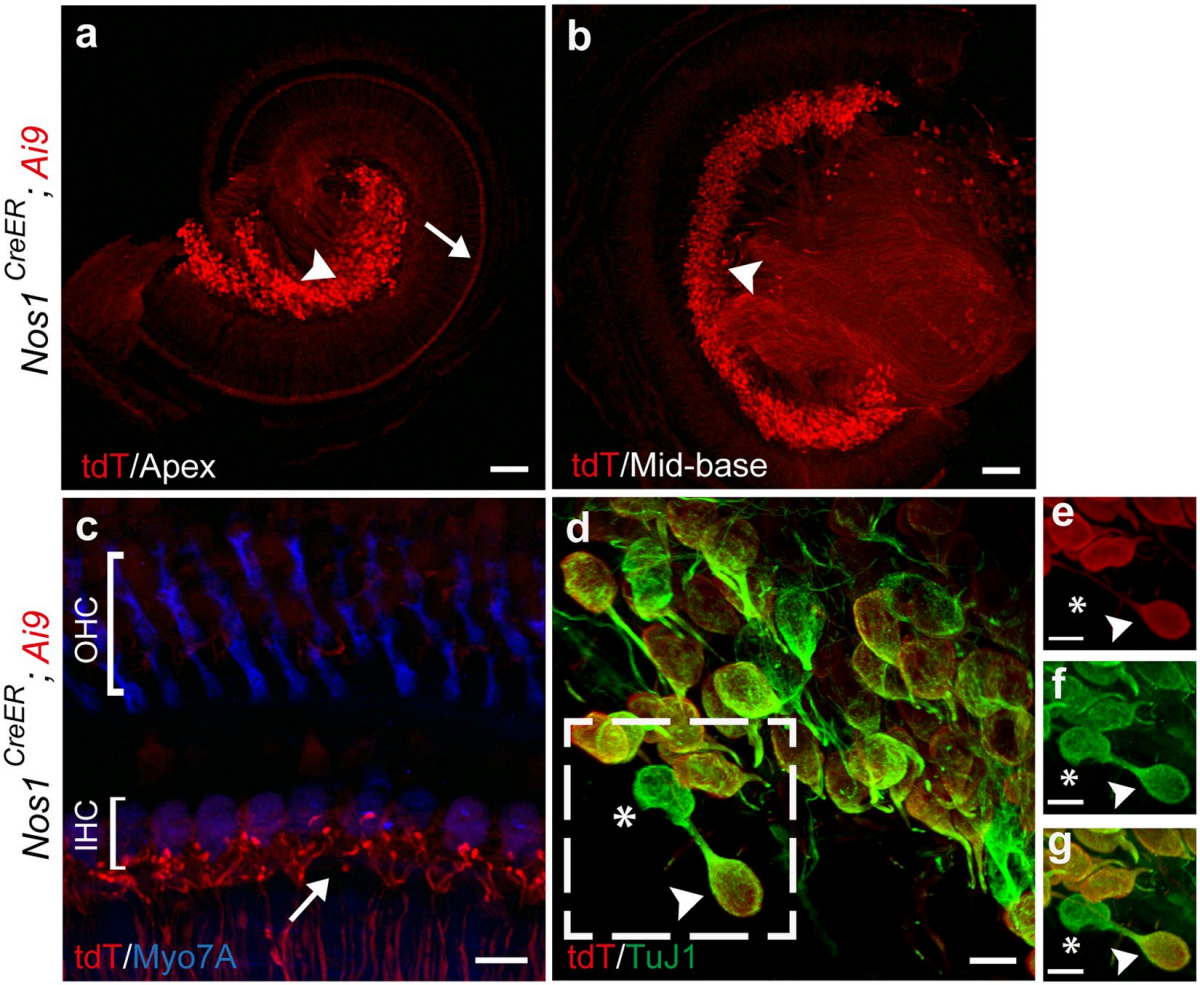
*Nos1*^*CreER*^ (neuronal nitric oxide synthase) specifically labels type I afferents. Cochlear whole mounts from apical turn (**a**) and mid-basal turn (**b**) of a 45-day old *Nos1*^*CreER*^*; Ai9* mouse show the expression of tdTomato (tdT, red) in spiral ganglion neurons (SGNs) (arrowheads) and in the inner spiral bundle (arrow in **a**). (**c**) Magnified view of the organ of Corti demonstrating tdTomato labeling in the bouton endings of type I afferent fibers contacting inner hair cells (IHCs) (arrow). Inner and outer hair cells are labeled blue with a Myosin VIIa (Myo7A) antibody. (**d**) Identity of *Nos1*^*CreER*^ positive SGNs (red, arrowhead indicating one example) is confirmed by co-labeling with TuJ1 (green). Asterisk indicates a small population of SGNs that do not express *Nos1*^*CreER*^. (**e**,**f**,**g**) Magnified images of the area marked by dashed outlines in **d**. (**e**) CreER expressing SGNs labeled by tdTomato antibody. (**f**) Type I SGNs labeled by TuJ1 antibody. (**g**) Merged image of **e** and **f**. Scale bars represent 100 μm (**a**,**b**), 10 μm (**c**,**d**) and 5 μm (**e**,**f**,**g**).

Nitric oxide is a gaseous neurotransmitter that has been implicated in many aspect of CNS function, including neuron structural plasticity, synaptic plasticity, regulation of blood flow and release of other neurotransmitters^13,14^. The expression of neuronal nitric oxide synthase (nNOS), the enzyme responsible for nitric oxide synthesis in neurons, was examined in pre-hearing (postnatal day (P)7–9) and hearing mice (P30–45) by crossing a knock-in *Nos1*^*CreER*^ mouse line with a tdTomato reporter line (*Ai9*). Upon induction with tamoxifen, the expression of reporter protein (tdTomato) was observed in SGNs throughout all cochlear turns (Fig. 1a,b). Upon closer examination of the organ of Corti, bouton endings of tdTomato-expressing SGNs were found in the IHC region (Fig. 1c) supporting their identity as type I afferent neurons that innervate IHCs. To investigate further the identity of the labeled neurons, co-immunolabeling was performed with β-tubulin 3 (TuJ1), which preferentially labels type I versus type II SGNs at young adult ages^15^. Most of the tdTomato-expressing SGNs were immunopositive for TuJ1 (Fig. 1d, also see Supplemental Video), confirming their identity as type I afferent neurons. It should be noted that since *Nos1*^*CreER*^ is an inducible Cre line, the recombinase efficacy is dependent on the dose of tamoxifen. We observed that a small fraction (~10%) of type I SGNs were not labeled (Fig. 1e,f,g) at the dose used in this experiment. Also, when the reporter expression of *Nos1*^*CreER*^*; Ai9* mice was induced with tamoxifen at pre-hearing ages (P2–5), a few non-neuronal cells were also found to express tdTomato in the osseous spiral lamina at P7 (Table 1) (see Supplemental Fig. S1), which were not observed when tamoxifen was injected after P10 and cochleas were analyzed between P30–45. As a control, *Nos1*^*CreER*^*; Ai9* mice without tamoxifen injection showed no labeling in the cochlea (see Supplemental Fig. S1). Immunolabeling for nNOS has been reported previously in different cell types in the cochlea, including but not limited to the inner and outer hair cells, SGNs and olivocochlear efferents^16–19^. However, the labeling pattern observed here was specific to the SGNs, and not found in hair cells or olivocochlear efferents. This discrepancy in the labeling patterns between nNos antibody and *Nos1*^*CreER*^*; Ai9* mice could be due to various factors, such as the lack of antibody specificity, low expression of CreER in other cell types at the time of tamoxifen induction, or the timing difference between the *Nos1* gene expression and nNos protein accumulation. The present results show that when induced in the second postnatal week, the *Nos1*^*CreER*^ mouse line can be used to label type I cochlear afferents specifically.

**Table 1:** Mouse models targeting type I versus type II SGNs.

Mouse line	SGN expression	Cochlear Region	Age Range Examined	Expression in other cell types	References
*Nos1* ^*CreER*^	Type I	All	P7-P45	Non-neuronal cells in the osseous spiral lamina observed when Tamoxifen injected between P2–P7	This work
*Th* ^2A-CreER^	Type II	Apex, mid	P7-P60	Lateral efferents	refs^11,12^
*CGRPα-EGFP*	Type II	Mid, base	P7-P45	Medial efferents, Lateral efferents, type I SGNs at prehearing ages	
*Slc6a4-GFP*	Type II	All	P7-P45	Platelets, Non-neuronal cells in stria vascularis	refs^26,27^
*Slc6a4* ^*Cre*^	Type II	All	P7-P45	Non-neuronal cells in the spiral osseous lamina and the stria vascularis	This work
*Drd2-Cre*	Type II	Mid, base	P7-P45	Sporadic labeling of apical medial efferents and lateral efferents	This work

### Serotonin Reuptake Transporter (*Slc6a4-GFP*) specifically labels type II cochlear afferents

**Figure 2:**
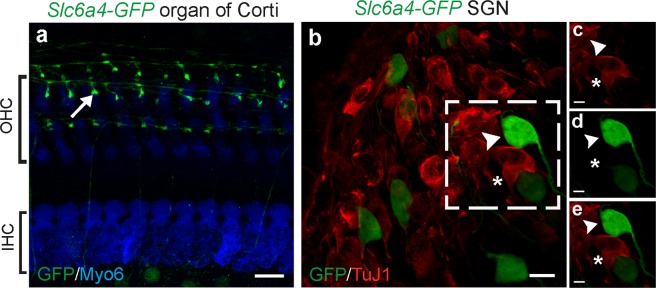
*Slc6a4-GFP* specifically labels type II SGNs. (**a**) Labeled type II afferent fibers (green) in the apical turn of a one-month-old *Slc6a4-GFP* mouse cochlea. Arrow indicates type II afferent boutons. Inner and outer hair cells are labeled with Myosin VI (Myo6) antibody (blue). (**b**) Antibody against TuJ1 (red) labels type I SGNs (asterisk) but not GFP-expressing type II SGNs (arrowhead). (**c**,**d**,**e**) Magnified images of the area marked by dashed outlines in **b**. (**c**) Type I SGNs labeled with TuJ1 antibody (red). (**d**) Type II SGNs expressing GFP (green). (**e**) Merged image of **d** and **e**. Scale bars represent 10 μm (**a**,**b**) and 5 μm (**c**,**d**,**e**).

Serotonin reuptake transporter (SERT) is a membrane protein encoded by the *Slc6a4* gene that recycles the neurotransmitter serotonin from the synaptic cleft into presynaptic neurons in a sodium-dependent manner^20^. In the auditory periphery, immunolabel of SERT has been reported in the olivocochlear efferent system^21^, auditory afferent fibers of developing marmoset^22^ and embryonic (E15.5) rat cochlear nucleus^23^. Serotonergic synaptic activity also has been demonstrated in the cochlea by the use of biochemical inhibitors^24^. Here *Slc6a4-GFP (also known as SERT-GFP)*, a BAC transgenic mouse line expressing GFP under the *Slc6a4* promoter was used to study the expression of SERT in the cochlea. Whole mount fluorescence microscopy of *Slc6a4-GFP* mouse cochlea (P30) showed the expression of GFP in fibers in the organ of Corti along the three rows of outer hair cells from which short branches with bouton endings connect with the OHCs; a pattern typical for type II afferents^1,11,25^ (Fig. 2a). When co-immunostained with β-tubulin 3 (TuJ1), GFP-expressing neurons did not overlap with TuJ1-positive type I SGNs (Fig. 2b–e), again supporting their identity as type II, but not type I, cochlear afferent neurons.

**Figure 3:**
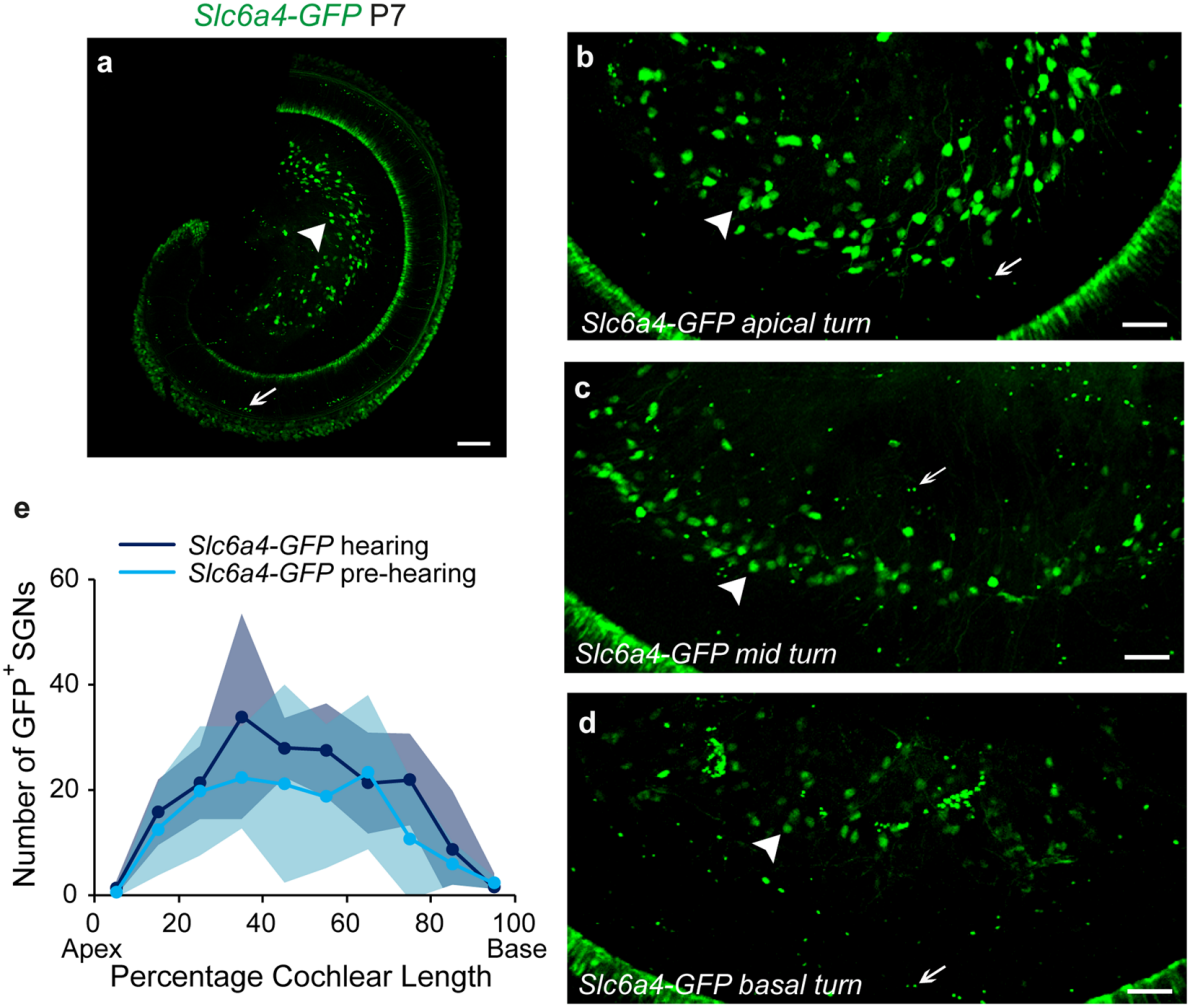
*Slc6a4-GFP* expression varies along the cochlea. Representative images from the apical turn (**a**,**b**), mid turn (**c**), and basal turn (**d**) showing the expression of *Slc6a4-GFP* in pre-hearing type II SGNs. Representative somata indicated by arrowheads, while platelets are indicated by arrows. Cochlear whole mounts from *Slc6a4-GFP* mice were analyzed before (P6–8, n = 5) and after the onset of hearing (P30–35, n = 6). (**e**) Each cochlear turn was divided into 10 bins of equal length along the cochlear spiral and the number of labeled SGNs in each cochlear bin were counted^12^. Shaded areas represent standard deviations. Scale bars represent 100 μm for all images.

The expression pattern of *Slc6a4-GFP* cochleas was examined in pre-hearing (P7–9) and hearing mice (P30–45). GFP-expressing SGNs were counted in bins by dividing the cochlear whole mounts into 10 segments along the tonotopic axis using ImageJ, as described previously^12^. Representative images of apical, mid and basal turns of cochlea with labeled SGNs in pre-hearing mice are shown in Fig. 3a–d. GFP-expressing neuronal somata (arrowheads point to example type II SGNs) were present in all cochlear turns at both ages. Numerous small-diameter cells (arrows) were observed in *Slc6a4-GFP* mouse cochleas at both ages that were easily distinguished from SGNs by their size. Since SERT is expressed in platelets^26,27^ and involved in regulating blood pressure^28^, these are likely to be platelets. Consistent with that conclusion, the putative platelets were found aligned within blood vessels (See Supplemental Fig. S3). The distribution of GFP-expressing SGNs peaks around 1/3 of the cochlear length from the apex and drops at both ends (Fig. 3e). Additional non-neuronal GFP-expressing cells were seen in the stria vascularis (Table 1).

Consistent with the observations using *Slc6a4-GFP* mice, another knock-in mouse line, *Slc6a4*^*Cre*^ (see Materials and Methods), also specifically labeled type II fibers when crossed with the *Ai9* reporter line and analyzed at ages between P7–45 (see Supplementary Fig. S4). Similar to *Slc6a4-GFP*, cochleas in *Slc6a4*^*Cre*^*; Ai9* mice also labeled non-neuronal cells in the osseous spiral lamina and stria vascularis, however, no expression was observed in  the platelets (Table 1) (see Supplementary Fig. S4). Finally, we examined the cochlear labeling pattern of *Slc6a4-Cre* BAC transgenic mice (see Materials and Methods). This line showed a less specific labeling pattern, that included expression in both type I and type II SGNs and cochlear efferents, and therefore the expression was not investigated further.

### *Drd2-Cre* mouse line labels type II afferents in the mid-basal region of the cochlea

**Figure 4:**
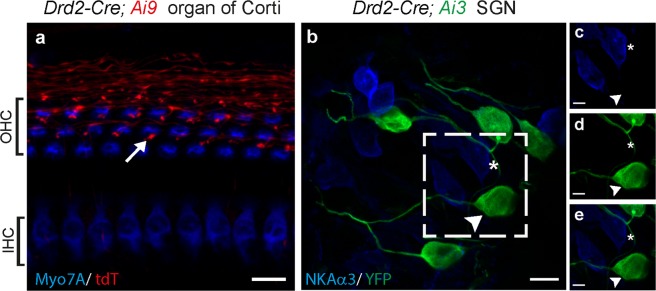
*Drd2-Cre* is expressed in type II cochlear afferents. (**a**) Cochlear whole mount of the mid turn of a one-month-old *Drd2-Cre; Ai9* mouse shows labeled type II afferent fibers (red) in the organ of Corti. Arrow indicates type II afferent bouton. Inner and outer hair cells are labeled with an antibody against Myosin VIIa (Myo7A, blue). (**b**) Co-immunolabeling with antibodies against α3 Na^+^/K^+^ ATPase (NKAα3, blue) suggests that *Drd2-Cre; Ai3* labeled SGNs (green) are not type I afferent neurons. (**c**,**d**,**e**) Magnified images of the area marked by dashed outlines in **b**. (**c**) Type I SGNs labeled with NKAα3 antibody (blue). (**d**) Type II SGNs immunoenhanced with GFP antibody (green). (**e**) Merged image of **c** and **d**. Arrowheads indicate SGNs labeled in *Drd2-Cre; Ai3* mouse cochlea. Scale bars represent 10 μm (**a**,**b**) and 5 μm (**c**,**d**,**e**).

Dopamine is a neurotransmitter of lateral olivocochlear efferents that regulate type I afferent signaling. Previous studies have reported the expression of dopamine receptor subtypes (D1–5) in spiral ganglion neurons by RT PCR and immunohistochemical analysis^29,30^. Type II-like morphology of fibers and their terminal boutons on OHCs could clearly be visualized in the mid and basal turns of *Drd2-Cre; Ai9* mouse cochleas at P30 (Fig. 4a). When *Drd2-Cre; Ai3* (*R26*^*LSL-EYFP*^) cochleas were co-immunostained with α3 Na^+^/K^+^ ATPase, which is expressed specifically in myelinated type I afferents and medial efferents but not in unmyelinated type II afferents and lateral efferents^31^, SGNs positive for EYFP (i.e., *Drd2-Cre* driven) or α3 Na^+^/K^+^ ATPase were mutually exclusive, as shown in the spiral ganglion region (Fig. 4b–e).

**Figure 5:**
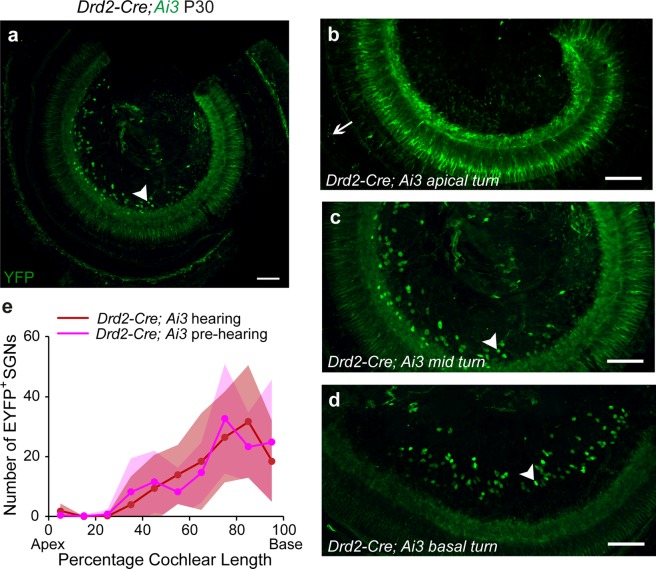
*Drd2-Cre* is expressed by type II afferents preferentially in the basal cochlea. Cochlear whole mounts of a P30 *Drd2-Cre; Ai3* mouse from the apical turn (**a**,**b**), mid turn (**c**) and basal turn (**d**) show the labeling of type II SGNs (arrowheads) and a few medial efferents (arrow in **b**). (**e**) Expression gradient of *Drd2-Cre* in mouse cochlea before (P6–8, n = 5) and after (P30–35, n = 5) the onset of hearing. Shaded areas represent standard deviations. Scale bars represent 100 μm for all images.

The expression pattern of *Drd2-Cre* was examined in pre-hearing (P7–9) and hearing mice (P30–45). Interestingly, the expression of *Drd2-Cre* was found only in the mid and basal type II afferents, although occasionally also in a few medial efferents in the apical cochlea (Fig. 5b, arrow) and presumably glia cells in the osseous spiral lamina (see Supplemental Fig. S5; Table 1). This expression gradient in type II SGNs across cochlea spiral is illustrated for pre-hearing cochlear whole mounts (Fig. 5a–d) and is similar for cochleas from hearing animals (Fig. 5e).

### Comparison of expression between different molecular markers for type II afferents

**Figure 6:**
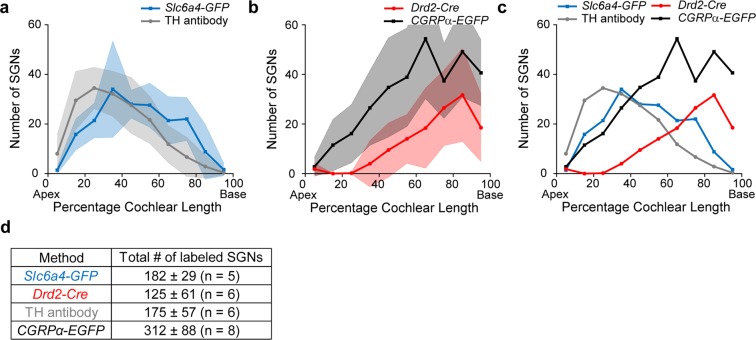
Comparative tonotopic distribution of molecular markers in type II afferents. (**a**) Comparative tonotopic distribution of type II neurons labeled in *Slc6a4-GFP* mice and by TH antibody immunostaining. (**b**) Comparison of the expression pattern of labeled type II neurons in *CGRPα-EGFP* and *Drd2-Cre* mice. (**c**) Average distribution of labeled type II SGNs using different methods. (**d**) Average total number of SGNs labeled by different methods. Age range of mice analyzed: TH immunostaining (P28-~P60), *Slc6a4-GFP* (~P30), *CGRPα-EGFP* (~P30), *Drd2-Cre* (~P30). All graphs show distribution patterns after hearing onset. Each cochlear turn was divided into 10 bins of equal length along the cochlear spiral and the number of labeled SGNs in each cochlear bin was recorded. Shaded areas represent standard deviations. TH immunostaining and *CGRPα-EGFP* mouse line data were reproduced from previous publication^12^.

Thus far, four molecular/genetic markers have been shown to label type II afferent neurons, described here or in Wu *et al*.^12^. To better illustrate and compare the tonotopic distribution of the labeled type II SGNs using these different strategies, graphical representations of these distributions at hearing age are shown in Fig. 6. Similar to *Th* and *Cgrpα (also known as Calca)*^12^, *Slc6a4-GFP* and *Drd2-Cre* also showed specific expression gradients along the cochlear coil (as summarized in Fig. 6c). Both TH antibody labeling as well as the distribution of *Slc6a4-GFP* labeled type II SGNs showed an apical preference, although the peak of TH labeling was found further apically (Fig. 6a). *Slc6a4-GFP* labeling is largely absent from the apical and basal extremes, but otherwise is distributed along the cochlear spiral, with a maximum at approximately 1/3 of the cochlear length from the apical end (Fig. 6a). Both *CGRPα-EGFP* and *Drd2-Cre* showed distributions biased towards the cochlear base. However, the *Drd2-Cre* labeled roughly half as many type II neurons compared to *CGRPα-EGFP* (P30, Fig. 6b). Given that the expression of *Cgrpα* is downregulated in type I SGNs during the first postnatal month^12^, there could be an overestimation for the number of type II SGNs based on *CGRPα-EGFP* labeling in one-month-old mice.

**Figure 7:**
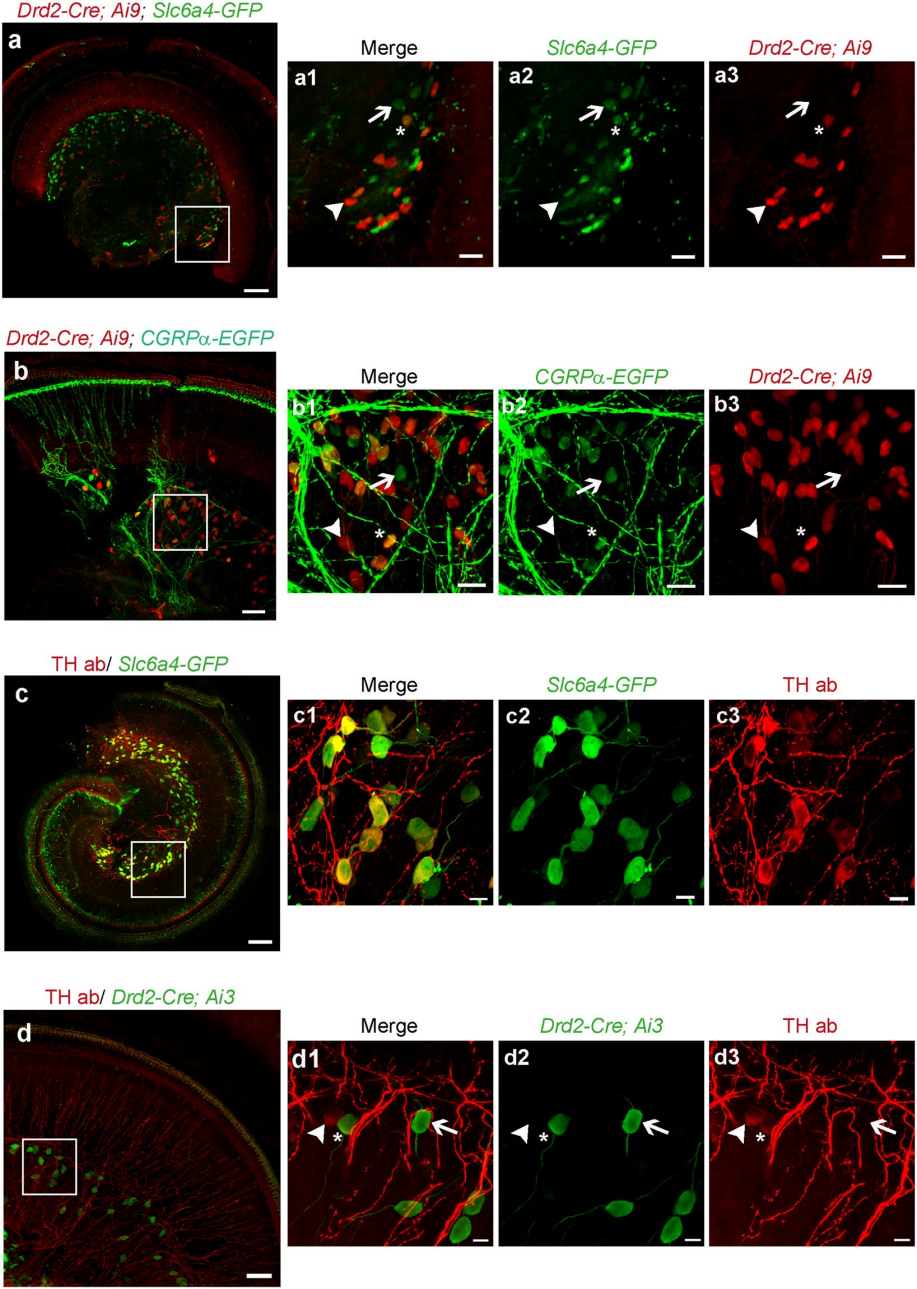
Co-expression of different molecular markers in type II afferents. (**a**) Organ of Corti from the mid turn of a triple transgenic mouse *Drd2-Cre; Ai9; Slc6a4-GFP* cochlea (P30). *Drd2-Cre* (arrowhead, red) and *Slc6a4-GFP* (arrow, green) can express individually or together in single neurons. (**a1**) Magnified area of inset from **a**, separated into individual channels in (**a2**,**a3**). Some SGNs were labeled by both mouse lines (asterisks, **a1**, **a2**, **a3**), some only by *Slc6a4-GFP* (arrows) and some only by *Drd2-Cre* (arrowheads). (**b**) *Drd2-Cre; Ai9; CGRPα-EGFP* can express individually or together in single neurons in the basal turn (**b1**) Magnified area of inset in (**b**), separated into individual channels in (**b2**,**b3**). Arrow indicates neurons expressing *CGRPα-EGFP* (green), arrowhead indicates neurons expressing *Drd2-Cre* (red) and asterisk indicates neurons co-expressing *Drd2-Cre* and *CGRPα-EGFP* (yellow). (**c**) SGNs in a P30 *Slc6a4-GFP* mouse cochlear apex labeled with TH antibody. *Slc6a4-GFP* (green) and tyrosine hydroxylase (TH, red) are co-expressed by most of the neurons in the cochlear apex and mid turn. (**c1**) Magnified area of inset in **c**, separated into individual channels in (**c2**,**c3**). (**d**) Organ of Corti from the mid turn of a transgenic mouse *Drd2-Cre; Ai3* (P7) immunostained with TH antibody. Although the labeled neurons are largely segregated *Drd2-Cre* (arrow, green) and TH *(*arrowhead, red) co-expression can occur in single neurons (asterisk). (**d1**) Magnified area of inset in **d**, separated into individual channels in (**d2**,**d3**). Scale bars represent 100 µm (**a**,**c**), 50 µm (**b**, **d**) 25 µm (**a1**–**a3**, **b1–b3**) and 10 µm (**c1–c3**, **d1–d3**).

To assess further type II afferent heterogeneity and to understand better how to utilize the different molecular and genetic markers for manipulating type II afferents, labeling patterns were compared at the level of individual SGNs. For example, two genetic markers with similar tonotopic distribution might be expressed by separate populations of type II SGNs. However, type II SGNs that co-express candidate genes were found in the overlapping cochlear regions. While type II SGNs expressing *Drd2-Cre; Ai9* and *Slc6a4-GFP* are largely segregated along the cochlear coil, individual SGNs co-expressing *Slc6a4-GFP and Drd2-Cre; Ai9* can still be found in the cochlear middle turn (Fig. 7a,a1,a2,a3 asterisk). *Drd2-Cre* and the previously reported *CGRPα-EGFP* mouse lines both label basal type II afferents. Cross-bred *Drd2-Cre; Ai9; CGRPα-EGFP* mice showed co-expression of the reporter proteins in some SGNs (asterisks in Fig. 7b,b1,b2,b3). ‘Apical’ reporters were examined in *Slc6a4-GFP* mouse cochleas labeled with antibodies against TH (Fig. 7c). Most of the type II SGNs in the cochlear apical region were co-labeled by both markers (Fig. 7c1,c2,c3). SGNs expressing TH and *Drd2-Cre* were largely restricted to apex or base, respectively. However, a few co-labeled SGNs could be found in the middle turn (Fig. 7d,d1,d2,d3, asterisk). Finally, as previous reported, TH and *CGRPα-GFP* expressing neurons could show co-expression in the middle turn of the cochlea^12^. We were not able to look for co-expression of *CGRPα-EGFP* and *Slc6a4-GFP*, because both mouse lines express the same reporter protein.

## Discussion

This work is part 3 of a series of studies to identify and validate mouse genetic tools for labeling and separately manipulating type I and type II afferents, the spiral ganglion neurons (SGNs) of the mammalian cochlea. As for previous studies in this series^11,12^ genetically modified mice obtained from commercial vendors and local laboratories were examined for expression of reporter proteins in cochlear afferents. This work describes a mouse with CreER coupled to the *Nos1* promoter that drove reporter expression specifically in type I but not type II SGNs. In addition, three mouse lines, *Slc6a4-GFP*, *Slc6a4*^*Cre*^ and *Drd2-Cre* could be used to target type II, but not type I, SGNs. Between tested genes, different apical-to-basal expression patterns and different amounts of overlap were found between markers, suggesting that subpopulations of type II neurons exist.

Does the labeling of specific groups of SGNs by these different mouse lines represent the endogenous expression pattern of these genes? *Nos1*^*CreER*^ was constructed by inserting CreER into the endogenous *Nos1* locus. Therefore, its expression most likely reflects the actual gene expression. The presence of nitric oxide synthase (NOS) has been described in cochlear tissue, including SGNs^17,18,32,33^. *Slc6a4-GFP* and *Drd2-Cre* were both made by random insertion of bacterial artificial chromosomes (BAC) containing the promoter and regulatory sequence of these two genes. Depending on where the BAC integrates in the genome, the expression may or may not reflect the endogenous pattern. However, the labeling patterns of these two lines have been validated in the central nervous system (CNS) by the GENSAT project (www.gensat.org). The specific expression of SERT in type II afferent neurons has been replicated with another *Slc6a4*^*Cre*^ knock-in line. For the *Drd2-Cre* line, the expression patterns in the CNS have been further validated by *in-situ* hybridization^34^. Additional supporting evidence comes from three recently published single-cell RNA sequencing (scRNAseq) studies on SGNs^35–37^. NOS1 was identified in all three studies as a gene that is expressed in type I but not type II SGNs. In addition, *Nos1* was expressed in all three subtypes of type I SGNs (based on principal component clustering), corresponding with the present observation of universal expression of reporter protein in type I SGNs. Similarly, TH and SERT were identified as marker genes for the type II SGNs in all three studies. Evidence for differential CGRPα expression in type II versus type I SGNs was reported in two of the studies. CGRPα was expressed at higher levels in type II SGNs than in type I SGNs^37^ and was among the genes expressed differentially by type I and type II SGNs^36^. *Drd2* expression was not reported in these publications, possibly due to low RNA levels, but the online data repository^37^ showed that *Drd2* is expressed at a low level in one of the subtypes of type I SGNs. The discrepancy between the scRNAseq result and the *Drd2-Cre* mouse line labeling could be due to various factors. It is possible that basal type II afferent neurons express *Drd2* during development and downregulate its expression in adults. Alternatively, because the *Drd2-Cre* mouse line is constructed by random insertion of BAC in the genome, which are prone to internal rearrangements, the reporter expression induced by this line may not represent endogenous expression of *Drd2* gene in the cochlea. To sum up, the expression patterns of NOS1, TH, CGRPα and SERT genes in SGNs in these transgenic mouse lines is largely consistent with scRNAseq data. We do see different types of unidentified cells in *Nos1*^*CreER*^, *Drd2-Cre* and *SERT-Cre* expressing mouse cochleas that were not traceable in the literature. These unidentified cell types were found in different regions of the cochlea, have different shapes and molecular foot prints and need further analysis for identification. Whether basal type II afferents express *Drd2* mRNA or protein awaits further investigation. It bears repeating that even without such confirmation, these mouse lines still can serve as experimental models for the study of SGNs.

Does the expression of these marker genes in the SGNs tell us anything about function? Nitric oxide regulates voltage-gated ion channels of hair cells^38,39^ and possibly can act as a retrograde transmitter to increase the probability of transmitter release from efferent terminals on inner hair cells prior to the onset of hearing^40^. Soluble guanylyl cyclase, the principal target of NO, is expressed in olivocochlear efferents^41^. Do the expression of TH, SERT and CGRPα suggest that type II afferent neurons use dopamine, serotonin and CGRP as neurotransmitters? Because they are all suggested olivocochlear efferent neurotransmitters^42^, previous studies of these neurotransmitters have focused logically on efferent neurons (in addition to glutamate transmission from hair cells) but made no mention of type II cochlear afferents. Dopamine release from lateral olivocochlear efferents can suppress the activity of type I afferents^43–45^ but the cellular effects of CGRP and 5-HT remain to be determined. Expression of TH is not in itself a guarantor of dopaminergic transmission. If the *Drd2-Cre* labeling represents the endogenous expression, the opposing patterns of *Th* and *Drd2* along the tonotopic axis further complicates any functional interpretation. Using RT-PCR, Drd2 receptor transcripts were identified in the OHCs^46^. Whether type II afferents could release dopamine in a retrograde fashion to act on OHCs requires further investigation. Thus, while these expression patterns may prove useful for future experimental strategies, they don’t change our present understanding of OHC to type II afferent synaptic function. Synaptic currents evoked in type II afferents by high potassium depolarization of cochlear tissue are due to glutamate release from outer hair cells that acts on AMPA^47–49^ and possibly kainate receptors^50^. Besides those synaptic signals, type II afferents respond to extracellular ATP with P2X and P2Y type receptors^4^. Intracellular recording from OHCs has yet to reveal any synaptic currents other than those due to acetylcholine release from cholinergic medial olivocochlear terminals^51–54^. If dopamine, or CGRP or 5-HT are released from type II afferent terminals in the cochlear nucleus, their actions there remain to be determined.

A hallmark of the cochlea is the ‘tonotopic’ organization of macro- and microscopic features that underlie acoustic frequency selectivity. For example, the basilar membrane increases in stiffness from apex to base, giving rise to the mechanically-tuned traveling wave described by von Békésy^55^. The neuronal innervation of the cochlea also varies along the tonotopic axis^56,57^. Afferent and efferent innervation is highest in mid regions of the cochlea (serving the most sensitive range of hearing) and there is a general tendency for greater numbers of both afferent and efferent contacts in the higher frequency cochlear base.

Since individual type I afferents contact a single inner hair cell in the mature cochlea, one might predict these neurons to be specialized according to their acoustic frequency selectivity. Indeed, there is already evidence supporting tonotopic variation of type I SGNs. For example, intracellular recording from dissociated type I afferents showed that basic membrane properties differed between those originating apically versus those from the cochlear base^58^. Moreover, within each of the three major subgroups of type I SGNs clustered by scRNAseq, there was tonotopic variation of expression for a subset of genes^35^. Taken together these results support the hypothesis that type I SGNs may fall into functional subgroups; with minor functional differences along the tonotopic axis.

Type II afferents are known to vary morphologically along the cochlear length^59^. Possibly due to the relatively small number of type II afferent neurons, very few studies have addressed heterogeneity among type II SGNs. Similarly, the recent scRNAseq studies also did not provide additional insights regarding subgroups within type II SGNs. However, expression patterns of different transgenic mouse lines have revealed ‘tonotopic’ heterogeneity among type II SGNs. Previous studies showed that promoters for TH and CGRPα were active in apical and basal type II afferents, respectively^11,12^. In the present work the promoters for SERT and DRD2 also drove tonotopic gradients in reporter protein expression by type II afferents. Each of these four genetic drivers had distinctive patterns of expression along the cochlea’s tonotopic axis. TH predominates in the apical half, SERT is expressed in a bell-shaped expression gradient with a bias toward the cochlear apex, while CGRPα and DRD2 appear preferentially in the cochlear base. In mid-cochlear regions of overlap, CGRPα and TH can be co-expressed by single type II neurons^12^. The present work also shows that single type II SGNs can co-express more than one label in regions of overlap, suggesting that these are not mutually exclusive populations. Rather, it suggests that the expression patterns found here reveal some underlying genetic differentiation related to cochlear position.

Why do type II afferents differ along the cochlea’s tonotopic axis? Comparisons to somatosensory afferents might be illuminating. Pain-sensing C-fibers of skin express CGRP and this signaling molecule plays a role in damage-triggered inflammation^60^. The preferential expression of CGRP by type II afferents in the higher frequency base of the cochlea would be consistent with the greater sensitivity of this region to acoustic trauma and so might serve an analogous function. In contrast, TH is expressed by unmyelinated low threshold mechanoreceptors (C-LTMRs) of skin^61^ that play a role in ‘emotional touch’^62^ and TH predominates in the low frequency cochlear apex where tissue trauma is less likely. It will be of interest to explore further the tonotopic diversity of type II afferents.

## Methods

All animal experiments were carried out in according to the guidelines approved by the Johns Hopkins Animal Care and Use committee (ACUC). Mice from both sexes were used in experiments. No obvious differences were observed between males and females in this study. For every finding, at least three experiments with animals from three litters were performed.

### Mouse Models

The mouse line *Drd2-Cre* [B6.FVB(Cg)-Tg(Drd2-cre)ER44Gsat/Mmucd] (RRID:MMRRC_032108-UCD) was bred on the C57BL/6 J background. It was generated by random insertion of a bacterial artificial chromosome (BAC) containing the regulatory sequences of *Drd2* gene followed by the Cre cassette as part of the GENSAT project^63^. The *Slc6a4-GFP* [Tg(Slc6a4-EGFP)JP55Gsat/Mmucd] (RRID:MMRRC_030692-UCD0) line, *Slc6a4-Cre* [Tg(Slc6a4-cre)ET127Gsat/Mmucd] (RRID:MMRRC_017261-UCD) and the *CGRPα-EGFP* [Tg(Calca-EGFP)FG104Gsat/Mmucd; RRID:MMRRC_011187-UCD] line were generated by GENSAT project using a similar strategy and obtained on mixed background. *Nos1*^*CreER*^ [B6;129S-Nos1^tm1.1(cre/ERT2)Zjh^/J]/(The Jackson Laboratories, #014541,) is on C57BL/6 J background and was generated by inserting a CreER^T2^ fusion gene into the *Nos1* locus. *Slc6a4*^*Cre*^ line [B6.129(Cg)-*Slc6a4*^*tm1(cre)Xz*^/J] (The Jackson Laboratories, #014554) is obtained on C57BL6 background and was generated by targeting a nuclear-localized Cre recombinase upstream of the first coding ATG of the *Slc6a4* gene. The Cre reporter lines *Ai3* [B6.Cg- Gt(ROSA)26Sortm3(CAG-EYFP)Hze/J, #007903] on C57BL6 background, *Ai9* [B6.Cg-*Gt(ROSA)26Sor*^*tm9(CAG-tdTomato)Hze*^/J, #007909] on C57BL6 background and *Ai32* [B6.Cg-*Gt(ROSA)26Sor*^tm32(CAG-COP4*H134R/EYFP)Hze^/J, #024109] on mixed background were purchased from The Jackson Laboratories.

### Tamoxifen Injection

Tamoxifen stock (Sigma #T5648) was prepared by dissolving tamoxifen in corn oil (Sigma #C8267) at a concentration of 10 mg/ml for sonication at room temperature (2 h). Stock solutions were stored at 4 °C in the dark and were used within 4 days of preparation. For studying the phenotype of postnatal day (P) 7 animals, tamoxifen was administrated through intragastric injection^64^ at P3 and P5 (0.2 mg each time) using an insulin syringe with an ultrafine needle (BD, 22 G). For analysis at 3–7 weeks, tamoxifen (1 mg) was administered by intra-peritoneal injections in the second postnatal week.

### Tissue Preparation and Immunofluorescence

Mice from postnatal day 5 to 40 were deeply anesthetized by isoflurane inhalation and decapitated. Temporal bones were removed and post-fixed in electron microscopy grade 4% paraformaldehyde (Electron Microscopy Sciences, Hatfield, PA) through the round and oval windows. The tissue was post-fixed for 1 h at room temperature (RT), rinsed with phosphate buffer solution (1X PBS), and dissected into apical, medial, and basal turns. Temporal bones from mice older than P25 were decalcified in 0.2 M ethylenediaminetetraacetic acid (EDTA) in PBS overnight at 4 °C after fixation. After rinsing with 1X PBS, cochlear turns were incubated in 30% sucrose for 10 min, permeabilized by quick freeze (−80 °C) and thaw (37 °C) and then washed with 1X PBS. The cochlear turns were then incubated in a blocking and permeabilizing buffer (10% normal donkey serum, 0.5% Triton X-100 in 1X PBS) for 1 h at RT. Primary antibodies were applied in incubation buffer (5% normal donkey serum, 0.25% Triton X-100 and 0.01% NaN_3_ in 1x PBS) for 48 hours at RT. Tissue was then rinsed in 1X PBS three times and incubated with Alexa Fluor-conjugated secondary antibodies (Molecular Probes) used at 1:1000 dilution for 1–2 h at room temperature. Cochlear tissue was rinsed three times with 1X PBS and mounted in FluorSave antifade mounting medium (CalBiochem, San Diego, CA). Primary antibodies used in this study include goat anti-GFP (1:5000, Sicgen #AB0020-200) rabbit anti-DsRed polyclonal antibody (1:1000, Takara #632496), mouse anti-NKAα3 (1:300, Thermo Fisher Scientific #MA3-915), mouse anti-TuJ1 (1:300, Biolegend #801201), rabbit anti-Myosin VI (1:500, Sigma-Aldrich #M5187), mouse anti-Myosin VIIa (1:200–500, DSHB #MYO7A), rabbit anti-TH (1:500, Millipore #657012-15UG), mouse anti-CD34 (1:50, BioLegend #343505).

### Image Acquisition and Quantification

Images were acquired on a LSM700 confocal microscope (Zeiss Axio Imager Z2) using 10× and 40× N.A. 1.30 oil immersion objectives. Images were processed using Fiji (RRID: SCR_002285), Photoshop CS6 (Adobe) and illustrator CS6 (Adobe). Quantification was carried out using Zen software (Zeiss) and Photoshop CS6 (Adobe). Quantification of spiral ganglion neuron numbers in each cochlear turn was performed as described previously^12^. Supplementary Video was made using syGlass system from IstoVisio Inc. (https://www.syglass.io/).

## Supplementary information

Supplementary data

Supplementary video 1

## Data Availability

Most of the data generated or analyzed during this study are included in this published article. All datasets from the current study are available from the corresponding authors on reasonable request.
