# Supplementary material for: Characterization of transgenic mouse lines for labeling type I and type II afferent neurons in the cochlea
Source: Sci Rep. 2019 Apr 3;9:5549. doi: 10.1038/s41598-019-41770-5 (PMC6447598; doi:10.1038/s41598-019-41770-5)
Supplement: Supplementary file 1 — Supplementary data [file 41598_2019_41770_MOESM1_ESM.docx]

**Characterization of transgenic mouse lines for labeling type I and type II afferent neurons in the cochlea**

Pankhuri Vyas^1,+^, Jingjing Sherry Wu^1, 2, +, #^, Adrian Jimenez^1^, Elisabeth Glowatzki^1, 2, *^ and Paul Albert Fuchs^1, 2, *^

^1^The Center for Hearing and Balance, Otolaryngology-Head and Neck Surgery, the ^2^Department of Neuroscience, Johns Hopkins University School of Medicine, Baltimore, MD, 21205, USA

# Current address: Department of Neurobiology, Harvard Medical School, Boston, MA 02115 USA

+ Both authors contributed equally

* Co-corresponding authors

**Address for correspondence:**

Paul Albert Fuchs and Elisabeth Glowatzki

The Johns Hopkins School of Medicine

Departments of Otolaryngology Head and Neck Surgery and Neuroscience

720 Rutland Avenue, Ross 818

Baltimore MD 21205, USA

Tel.: 410-955-6311

Fax: 410-614-4748

Email: pfuchs1@jhmi.edu, eglowat2@jhmi.edu


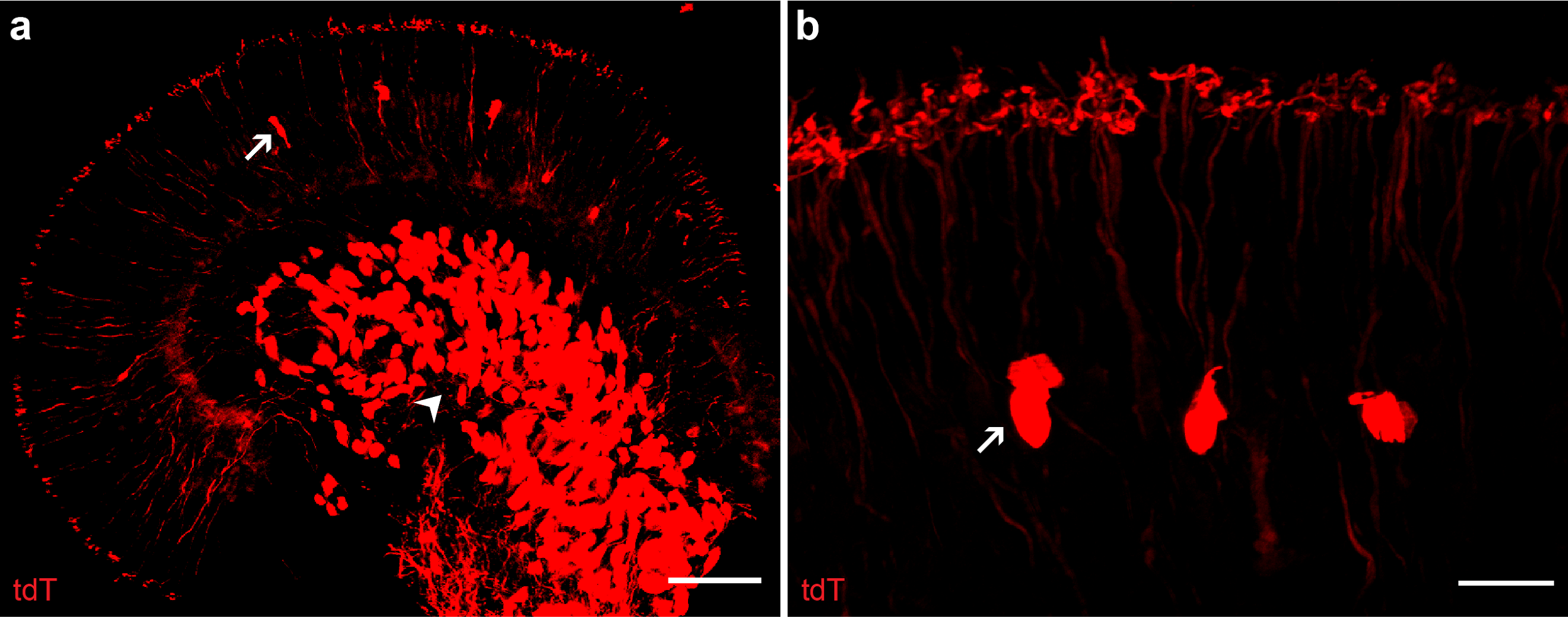


**Supplemental Figure S1**

1. Whole-mount of the apical turn cochlear epithelium from a P7 *Nos1^CreER^; Ai9* mouse, demonstrating the labeling of SGNs (arrowhead) and non-neuronal cells at the osseous spiral lamina (arrow). Scale bar 100 µm.
2. A higher magnification image of a P7 *Nos1^CreER^; Ai9* mouse cochlea middle turn, demonstrating the morphology of the non-neuronal cells (arrow). Scale bar 20 µm.


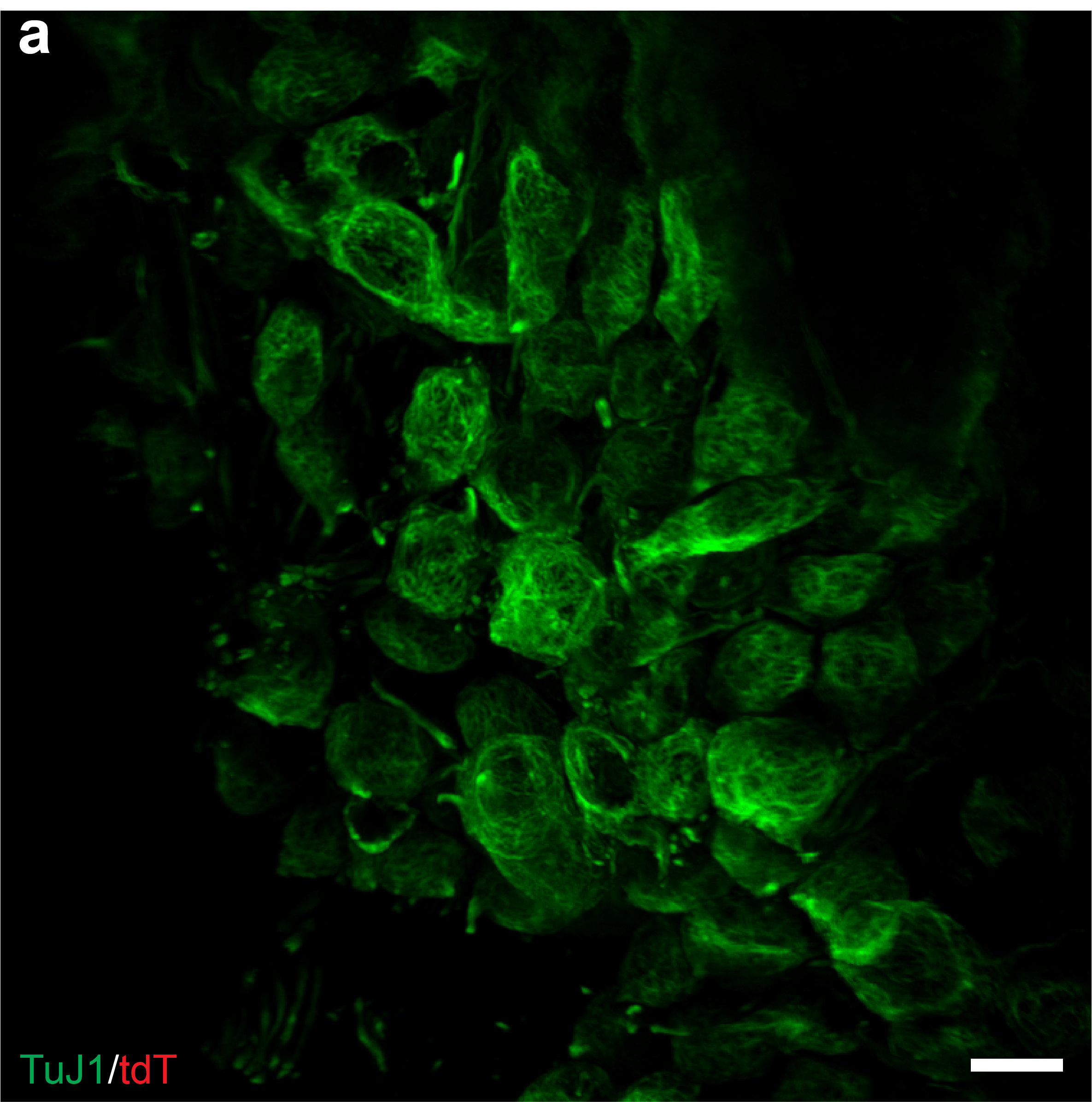


**Supplemental Figure S2**

1. Whole-mount of the cochlear middle turn from a P30 *Nos1^CreER^; Ai9* mouse, which was not administered with tamoxifen, demonstrating that there is no leaky expression of reporter protein (tdTomato) in the cochlea. Type I SGNs are labeled with TuJ1 (green). Scale bar 10 µm.


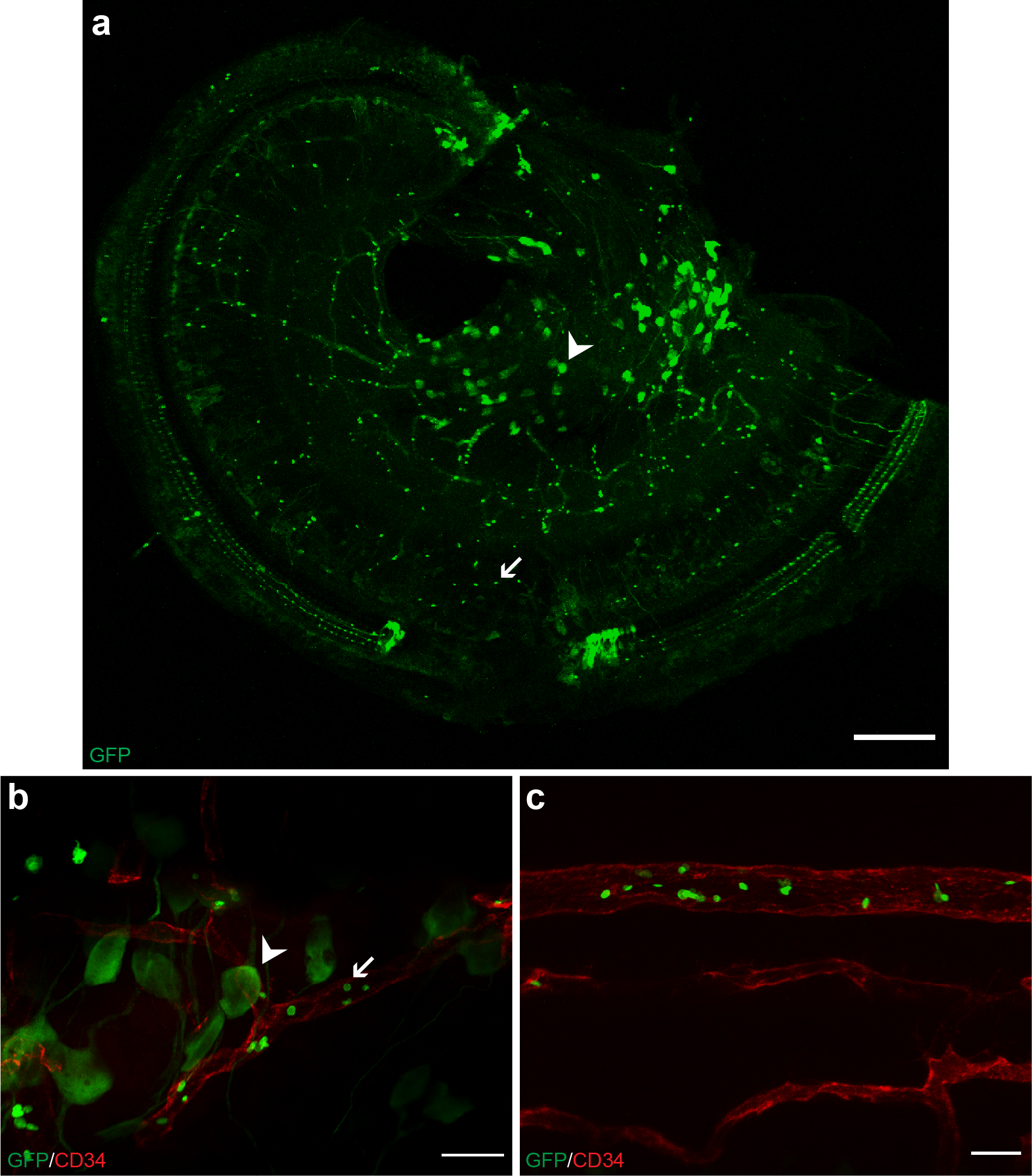


**Supplemental Figure S3**

1. Apical turn of a P30 *Slc6a4-GFP* mouse cochlea. Both larger diameter SGNs (arrowhead) and smaller diameter cells (arrow) are labeled in this mouse line. Note that the smaller diameter cells follow the meandering path of the blood vessels. Scale bar 100 µm.
2. A higher magnification image of the modiolar region of the apical turn of a P25 *Slc6a4-GFP* mouse cochlea, demonstrating labeled SGNs (arrowhead) and smaller diameter cells (arrow). The smaller diameter cells possibly are platelets, because they are contained within the blood vessels immunolabeled with CD34. Scale bar 20 µm.
3. Another example demonstrating the putative platelets labeled in a P5 *Slc6a4-GFP* mouse cochlear middle turn. Scale bar 15 µm.


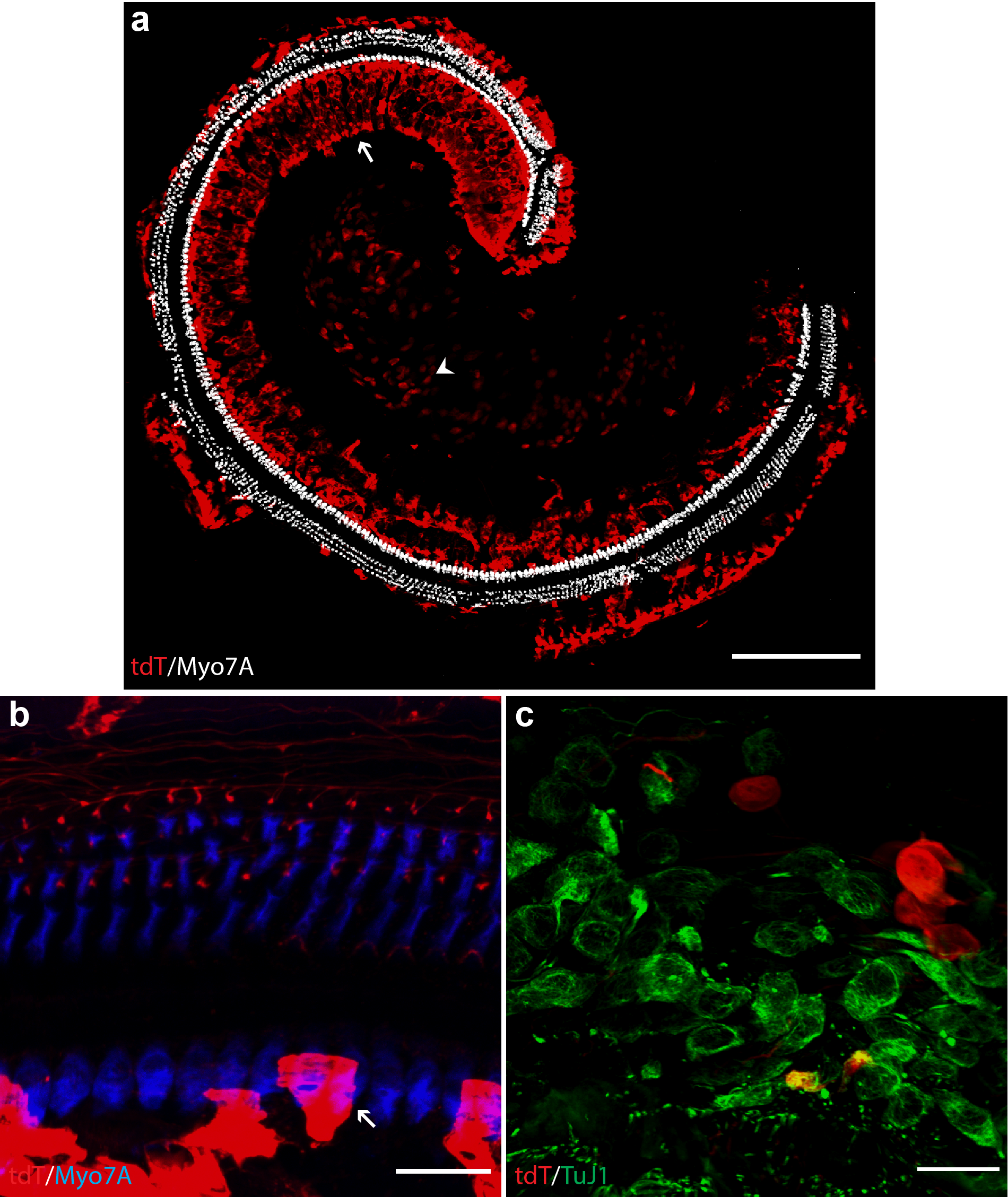


**Supplemental Figure S4**

1. Representative image of a P17 *Slc6a4^Cre^;Ai9* mouse cochlea apical turn, demonstrating the labeling of SGNs (arrowhead) and non-neuronal cells in the osseous spiral lamina (arrow) and the stria vascularis. The hair cells were labeled by antibodies against Myosin VIIa. Note that only the hair cell region of the Myosin VIIa immunolabeling was included in this image, to better visualize the modiolar region of the cochlea. Scale bar 200 µm.
2. Organ of Corti from P17 *Slc6a4^Cre^;Ai9* mouse cochlea apical turn shows the labeling of type II afferent fibers that innervate the OHC region but not the IHC region. Note non-neuronal cells labeled near IHC region (arrow). Scale bar 20 µm.
3. Spiral ganglion region of P45 *Slc6a4^Cre^;Ai9* mouse cochlea immunolabeled with TuJ1 and tdTomato antibodies. tdTomato-positive neurons are not labeled by TuJ1 antibody, an adult type I SGN marker, demonstrating that *Slc6a4^Cre^* specifically labels type II afferent neurons. Scale bar 20 µm.


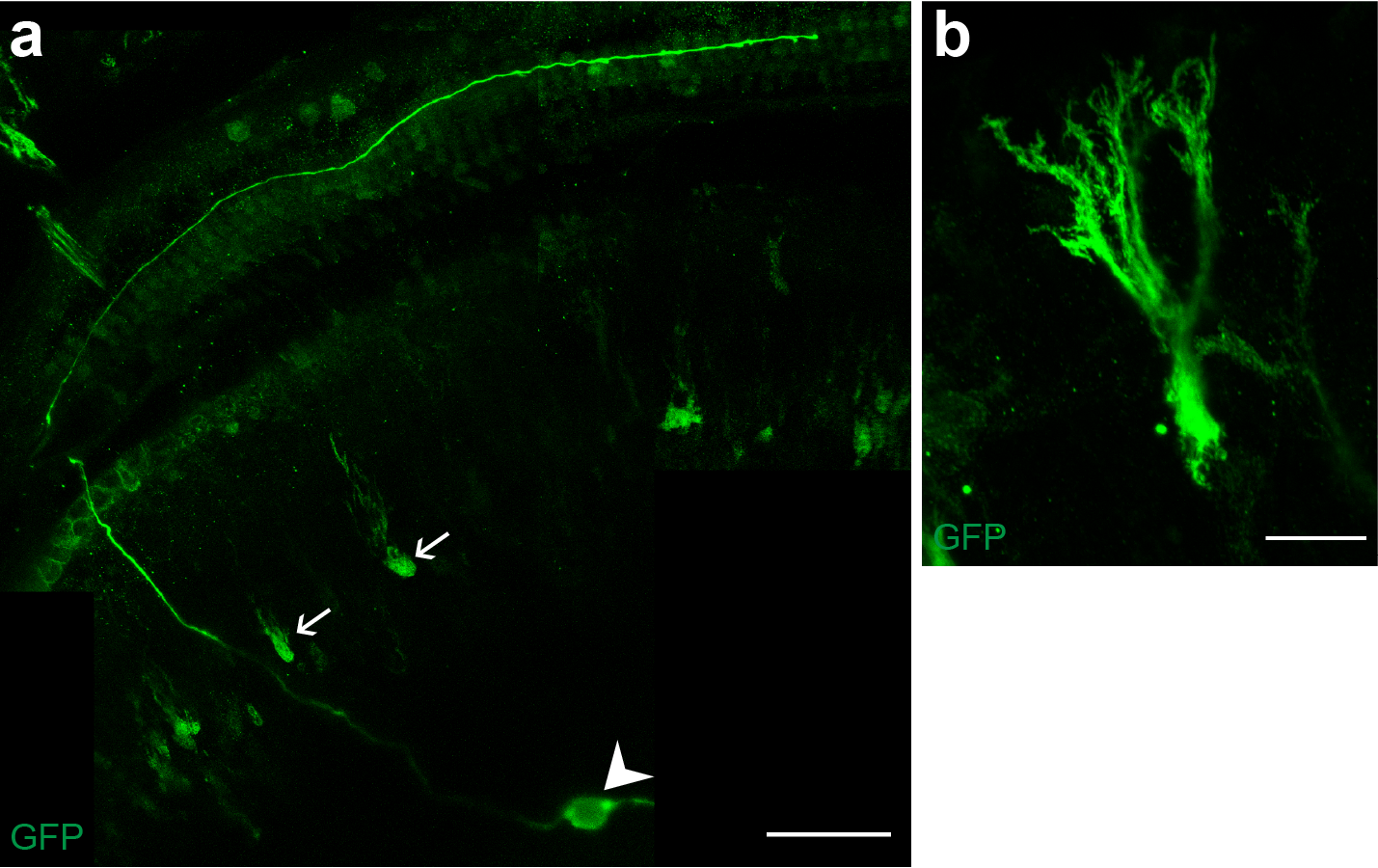


**Supplemental Figure S5**

1. Representative image taken from the basal turn of a P13 *Drd2-Cre; Ai32* mouse cochlea. Mouse line labeling was immuno-enhanced by antibody against GFP. Arrowhead indicates a labeled type II SGN that extends a single dendrite to OHCs. Arrows indicate unidentified cells in the osseous spiral lamina. Scale bar 50 µm.
2. Higher magnification image taken from the osseous spiral lamina of a P37 *Drd2-Cre; Ai32* mouse cochlea apical turn, detailing the morphology of a labeled unidentified cell. Scale bar 10 µm.
